# The role of osteoprotegerin in the development, progression and management of abdominal aortic aneurysms

**DOI:** 10.1515/med-2020-0046

**Published:** 2020-05-26

**Authors:** Maciej Migacz, Agata Janoska-Gawrońska, Michał Holecki, Jerzy Chudek

**Affiliations:** Department and Clinic of Internal, Autoimmune and Metabolic Diseases, Faculty of Medicine, Medical University of Silesia in Katowice, Poland; Department and Clinic of Internal Medicine and Cancer Chemotherapy, Faculty of Medicine, Medical University of Silesia in Katowice, Poland

**Keywords:** atherosclerosis, aortic aneurysm, management, osteoprotegerin, prognosis

## Abstract

Osteoprotegerin (OPG) appears to be a very promising marker both in the diagnosis of abdominal aortic aneurysms (AAAs) and as a potential target in its treatment. This article presents an overview of the current literature that discusses the role of OPG in the pathogenesis of atherosclerosis and its potential value as a prognostic factor in AAA. Pharmacological modulation of OPG expression has been considered. In conclusion, it seems that further research designed to assess the relationship between OPG and AAA is needed as this may contribute to improved AAA monitoring and more effective treatment of patients with AAA.

## 1 Introduction

Aortic aneurysm is, after atherosclerosis, the second most common aortic condition and a major cause of morbidity and mortality [1]. The prevalence and incidence of abdominal aortic aneurysms (AAAs) increase with age and are especially higher in men (in approximately 2% of the male patients aged over 65 years) [2]. As a progressive disease, AAAs tend to gradually enlarge and eventually rupture in an individually variable manner. Aneurysm rupture is the main complication of AAA and is fatal in nearly 80% of the cases [3]. The main hallmarks of AAA pathogenesis include chronic inflammation (accompanied by macrophage accumulation on the aortic wall), apoptosis of vascular smooth muscle cells (VSMCs), degradation of extracellular matrix and thrombosis in arterial plaques, which leads to deficits in oxygen perfusion (persufflation) to the arterial wall [4]. Various proinflammatory cytokines have been shown to stimulate the activity of proteolytic enzymes on the aortic wall [5], including tumor necrosis factor-α (TNF-α), osteopontin (OPN) and osteoprotegerin (OPG), which appears to be a very promising marker in the diagnosis and as the target in the therapy of AAA. Radical treatment, either surgical or endovascular, has been considered to be the only effective management of a large AAA. However, pharmacotherapy to inhibit AAA progression and thereby reduce the risk of aneurysm rupture is being actively explored. Recent data show that the treatment of AAAs generates substantial healthcare costs worldwide [6]. There is an opportunity to considerably reduce the costs of patient care in AAAs by careful follow-up and more effective treatment.

This article presents an overview of the current literature that discusses the role of OPG in the pathogenesis of atherosclerosis and its potential value as a prognostic factor in AAA.

## 2 The characteristics of OPG

OPG is a glycoprotein cytokine receptor identified in 1997 by Simonet et al. and belongs to the TNF receptor superfamily (TNFRSF11B) [7]. Jurisic et al. reported that an elevated concentration of TNF-α correlates with the number of inflammatory cells and the degree of vascularization [8]. Furthermore, TNF-α has been associated with the progressive inflammatory process that promotes aneurysm progression [9]. Elevated levels of TNF-α were observed in patients with AAA, suggesting that TNF-α plays a central role in the regulation of matrix remodeling and inflammation in the aortic wall, leading to the development of AAA. In addition, TNF-α deficiency attenuates matrix metalloproteinase-2 (MMP-2) and MMP-9 expression and macrophage infiltration into the aortic tissue.

OPG is a soluble “decoy receptor” for the receptor activator of nuclear factor kappa B ligand (RANKL) and TNF-related apoptosis-inducing ligand (TRAIL) [7,10,11]. The RANK/RANK ligand/OPG pathway has been shown to be involved in bone remodeling and to modulate the differentiation and activation of osteoclasts, thereby influencing the balance between bone formation and resorption [12]. RANK is found on the surface of osteoclast precursors, including monocytes, macrophages and dendritic cells [13,14]. While RANKL is expressed on the surface of stromal cells, osteoblasts and T cells [15], OPG binds to RANKL to competitively inhibit the interaction between RANKL and its receptor, which translates into a bone protective action [16]. Some studies suggest that OPG is not only a bone protector but also acts as a protective factor for the cardiovascular system. Moreover, OPG may become the new biomarker of cardiovascular diseases and atherosclerosis in humans [17,18,19]. Evidence suggests that OPG is associated with the development of peripheral artery disease, coronary and cerebrovascular atherosclerosis, endothelial damage [20], aortic aneurysms, valvular heart diseases [21] and heart failure in patients with a history of myocardial infarction [22]. Additionally, Wajda et al. showed that serum OPG levels are a significant and independent predictor of death in patients with stroke assessed on admission to the stroke unit [23]. The increase in OPG plasma concentrations in patients with arteriosclerosis is an expected response to intensified inflammatory activation. OPG is expressed on vascular walls, as well as in VSMCs and endothelial cells [24]; OPG release can be modulated by proinflammatory cytokines, including interleukin-1β and TNF-α [25,26]. OPG was also found to stimulate the secretion of extracellular matrix metalloproteinases (MMP-2 and MMP-9) through the mediation of monocytes, endothelial cells and VSMCs of the aorta [27], which in turn contributes to the damage of perivascular fibrous capsule and plaque instability (Tables 1 and 2).

**Table 1:** Participation of OPG in the pathogenesis of abdominal aortic aneurysms

Progression		Protection	
Character	Ref.	Character	Ref.
Activation of inflammatory mechanisms (modulated by proinflammatory cytokines including interleukin-1β and TNF-α)	[24], [25], [26]	Proliferation of VSMCs	[31], [32]
Increased proteolysis in the vascular wall (by stimulating monocytes, endothelial cells and VSMCs to secrete extracellular matrix metalloproteinases [MMP-2/9])	[27], [33]	Increased collagen production in atherosclerotic plaques	[31]
Apoptosis of VSMCs	[27]		

**Table 2:** Osteoprotegerin (OPG) and progression of abdominal aorta aneurysms (AAA)

↑OPG and incidence AAA	[27], [33], [40], [41]
No correlation between OPG and incidence of AAA	[42]
↓OPG, ↓dilatation and risk of aorta rupture	[50]
↓OPG, ↑dilatation and risk of aorta rupture	[52]
↓OPG expression, ↓dilatation and risk of aorta rupture	[51]
↑OPG expression, ↓dilatation and risk of aorta rupture	[53]

The double inactivation of OPG^−/−^ and apolipoprotein-E^−/−^ (ApoE) in studies on mice was shown to accelerate the progression of arteriosclerotic lesions and vessel fibrosis compared to the loss of ApoE [28] alone. These findings confirm the results of earlier research in which increased large artery fibrosis was observed, including the proliferation of intima and media in mice with targeted loss/disorders of OPG [29]. In line with these discoveries, the inactivation of OPG expression in the transgenic OPG^−/−^ mice prevented the development of calcified lesions in the arteries of mature mice [30]. It was also demonstrated that elevated serum OPG concentrations were linked to the increased count (proliferation) of VSMCs and collagen production in plaque, although plaque size, vascularization and the intensity of inflammatory condition in the arteriosclerotic lesions remained unaffected [31]. This was confirmed in a study by Candido et al. [32], in which a 12-week exposure to recombinant OPG in apoE-null mice was found to be associated with a small increase in the total aortic plaque area but marked increase in the number of plaque smooth muscle cells in comparison to that in vehicle-treated animals at necropsy. There was no difference in the number of collagen fibers and the degree of plaque macrophage infiltration between treated (with OPG) and untreated animals.

These findings speak of the beneficial effect of OPG on the formation and stability of atherosclerotic plaque, which may prevent or slow the progression of AAA.

## 3 OPG concentrations versus AAA

Elevated concentration of OPG in AAA was initially reported by Moran et al. in 2005 [27]. Increased OPG levels were observed in aortic wall explants in patients with AAA compared with biopsies of the atherosclerotic narrowed aorta. Also, an *in vitro* study revealed that recombinant human OPG stimulates the activity of MMP-9 in aortic VSMCs and thereby limits cell proliferation and survival; it was also shown to induce IL-6 secretion and MMP-2/9 activity in monocytes, which play an even more clear role than OPG in AAA formation. Koole et al. analyzed the relationship between OPG concentrations in biopsy specimens of AAAs and the aneurysm diameter adjusted for cardiovascular risk factors (age, sex, arterial hypertension, diabetes, smoking, chronic obstructive pulmonary disease and a history of myocardial infarction) [33]. OPG concentrations were shown to be correlated with larger AAA diameter, adjusted for the relevant confounders. A correlation was also present between MMP-2 and MMP-9 activities and OPG concentration, which plays an important role in the remodeling of walls in AAA, as previously mentioned.

AAA patients are extensively treated with statins, since statins are supposed to reduce cardiovascular mortality in patients with cardiovascular risk factors and following abdominal aneurysm repair [34,35]. The available data (performed both *in vitro* and *in vivo*) suggest that HMG-CoA reductase inhibitors may slow the progress of AAA by inhibiting the MMP activity in the aortic wall [36,37]. Statin therapy is therefore recommended in the conservative treatment of patients with AAA to slow down the growth of aneurysms [38]. Muehling et al. investigated the relationship between statin use and the concentrations of MMP-2, MMP-9, OPG, IL-6 and IL-10 in biopsy specimens of aortic walls in patients with AAA, but found no correlation with OPG concentrations [39]. As the effectiveness of statin therapy in AAA management remains uncertain, further clinical trials with long-term follow-up are needed.

A further study investigated the relationship between pulse wave velocity (PWV), a measure of arterial stiffness, and serum OPG concentrations in AAA patients [40]. A significant increase in PWV and OPG concentrations was observed in patients with AAA compared to the controls with normal aortic diameter. The same authors evaluated PWV in patients who underwent endovascular AAA repair [41]. PWV and OPG levels were compared in patients with two different graft coatings, either polytetrafluoroethylene (PTFE) or polyester. As in the previous study, both elevated PWV and serum OPG concentrations were observed in patients with AAA. It is worth noting that increase in PWV was greater in patients with polyester-covered endografts compared to PTFE coating. Serum OPG concentrations were found to be decreased in both groups; however, the decrease was more pronounced in patients with PTFE-covered endografts. Serum OPG concentrations in patients with AAA > 50 mm following open versus endovascular aortic aneurysm repair (34 vs 40 patients, respectively) were analyzed by Filis et al. [42]. Patients with a history of inguinal hernia repair, but without AAA, acted as controls. Interestingly, no correlation was found between OPG concentration and the presence or extent of the aneurysm. One may hypothesize that serum OPG concentration reflects the intensity of atherosclerosis across vasculature rather than in the aneurysm wall alone. In other words, further research is necessary to determine the possible value of OPG concentrations in detecting and monitoring the progression of AAA.

**Figure 1:**
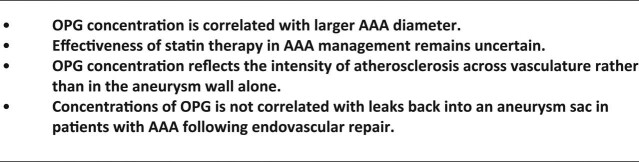
OPG concentrations versus AAA.

A leak back into an aneurysm sac is a relatively frequent event in patients with AAA following endovascular repair, and this complication can only be diagnosed by long-term follow-up including regular imaging of the aorta. It is worth noting that Moxon et al. diagnosed this type of complication in patients with the largest diameter aneurysms (in 24 of 75 patients) [43]. The authors analyzed MMP-9 activity and serum concentrations of OPG, d-dimer, homocysteine and C-reactive protein as the possible indicators of leaks in patients with AAA undergoing endovascular aneurysm repair. Regrettably, none of the tested indicators was confirmed to be correlated with leaks in any of the study groups (Figure 1).

## 4 Drug-induced modulation of OPG expression

The diameter of AAA is an important clinical risk factor of aneurysm rupture and is routinely used in qualifying patients for repair procedures [44]. All AAA treatment methods are primarily intended to limit the risk of the major complication, which is an aneurysm rupture. The drug modulation of OPG expression is currently the subject of many studies intended to improve prognosis in patients with AAA. So far, studies on humans and animals have suggested that the phenotype of smooth muscle cells in aortic aneurysms may be conducive to the degeneration of the aortic extracellular matrix [45,46,47,48,49].

Moran et al. [50] used ApoE^−/−^/OPG^−/−^ dual inactivation in angiotensin II (Ang II)-induced aortic aneurysms in mice. OPG deficiency was shown to limit aortic dilatation and the rupture risk of aortic aneurysm induced by Ang II. Complementary evidence has been submitted suggesting that OPG significantly contributes to aneurysm wall weakening. OPG deficiency was demonstrated to have no effect on the hypertensive response to Ang II infusion, which may suggest that the mechanisms of aortic aneurysm formation involving OPG are independent of blood pressure. Lower levels of proinflammatory cytokines (TNF-α, IL-6 and MCP-1) were also observed as well as reduced cathepsin S concentrations and lower MMP-2/MMP-9 activity in conditions of OPG deficiency following Ang II infusion. The phenotype of aortic smooth muscle cells was also analyzed to reveal that OPG deficiency promotes a more stable smooth muscle cell phenotype in the aortic wall (demonstrating lower expression ratio of apoptosis markers Bax/Bcl2, which was also observed in the aortas of ApoE^−/−^/OPG^−/−^ mice) as compared to the phenotype more conducive to the degeneration of extracellular matrix. In conclusion, it is considered that OPG deficiency reduces enhanced proteolysis in aortic walls and inflammatory phenotype induction in aortic VSMCs in response to Ang II, thereby reducing aortic dilatation and the risk of rupture.

A study of sclerostin (a protein involved in the regulation of bone metabolism) provided interesting insights into OPG expression [51]. It was demonstrated that transgenic introduction of human sclerostin gene (SOST) and exposure to recombinant mice sclerostin in mice with ApoE^−/−^ deficiency slowed down the formation of atherosclerosis and Ang II-induced AAA. Limited extracellular matrix degeneration, reduced elastin breaks and preserved collagen structure in aortic walls were observed in both study groups. Importantly, reduced OPG expression in aortic walls was confirmed in mice following transgenic SOST introduction, attributed to Wnt pathway inhibition (wingless-type mouse mammary virus integration site). These findings are consistent with reduced SOST expression reported in an *in vitro* study of human AAA specimens.

OPG was shown to have an opposite effect on the formation of AAA in a study by Bumdelger et al. [52] OPG inactivation in mice with CaCl_2_-induced AAA was found to induce the development of aneurysm. In another study, a team of researchers (Kamata, Bumdelger et al.) demonstrated a beneficial inhibitory effect on the progression of AAA following oral administration of eicosapentaenoic acid (EPA) in mice with OPG inactivation [53]. EPA reduced the phosphorylation of both TAK-1 and JNK and also decreased MMP-9 expression by Gpr-120/Ffar-4 in aortic VSMs in CaCl_2_-induced AAAs. This finding may suggest that OPG exhibits a protective action on aortic walls and that EPA action may be used in conditions of OPG deficiency.

**Figure 2:**
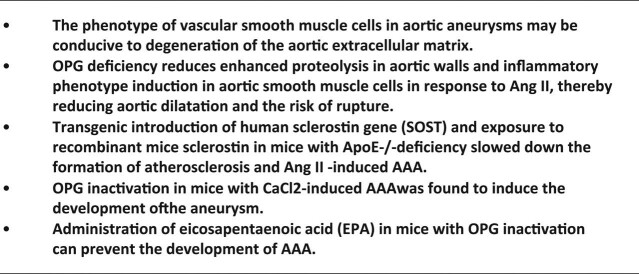
Drug-induced modulation of OPG expression.

It is unclear, however, whether OPG alone can prevent the development of AAA (Figure 2).

## 5 Use of OPG in the treatment of AAA

A recently published study in an animal model analyzed the protective action of small OPG doses in the treatment of AAAs induced by Ang II (upstream of the renal arteries, where thrombus may develop) and CaCl_2_ (downstream of the renal arteries, without thrombus formation) [54]. A different AAA location is attributed to the difference in the composition of collagen and elastin in these sections of the abdominal aorta. Exposure to human recombinant OPG (1 µg 2×/week, 2 weeks before AAA induction) did not slow down Ang II-induced AAA formation in ApoE^−/−^ mice. This finding suggests that exogenous OPG does not contribute to the inhibition of AAA formation in ApoE^−/−^ mice, unlike atherosclerosis-inhibiting endogenous OPG [28]. A significant increase in aortic wall thickness and collagen content in aortic walls was observed in another model of CaCl_2_-induced AAA, following administration of human recombinant OPG. In addition, no cases of aneurysm rupture were observed, which were reported in the Ang II-induced AAA model. This may suggest that OPG has a protective effect against AAA rupture in an advanced stage of the disease. On the other hand, the progression of aneurysm, the inflammation of aortic wall and the remodeling of the extracellular matrix and aortic VSMCs continued, which was also the case in the Ang II-induced AAA model. As a result, AAA rupture models and higher OPG doses should be investigated to determine whether OPG has any protective action against the risk of aneurysm rupture.

## 6 Summary and conclusion

It appears that there is a close relationship between OPG and AAA. However, further research is needed to investigate the mechanisms of the aneurysm development and the action of pharmaceuticals in order to come up with improved treatment strategies for patients with aortic aneurysm. The therapeutic potential of OPG expression modulation, showing a beneficial attenuating effect on AAA formation in animal models, is high and is the subject of continuing research. This article has reviewed the potential benefits of OPG-level measurements as a biomarker for monitoring the size of AAA. However, serum OPG concentrations in patients following surgical AAA repair have not been useful as an indicator of AAA rupture.

In conclusion, it seems that further research designed to assess the relationship between OPG and development of AAA may improve monitoring and effectiveness of the aneurysm therapy.
